# The emotion prediction of college students with attention LSTM during the COVID19 epidemic

**DOI:** 10.1038/s41598-023-50322-x

**Published:** 2023-12-20

**Authors:** Mengwei Wu, Shaodan Lin, Chenhan Xiao, Xiulin Xiao, Siwei Xu, Shuhan Yu

**Affiliations:** College of Mechanical and Intelligent Manufacturing, Fujian Chuanzheng Communications College, Fuzhou, 350007 China

**Keywords:** Human behaviour, Computer science

## Abstract

During the COVID19 pandemic, there is a pronounced collective mental health issue among college students. Forecasting the trend of emotional changes in on-campus students is crucial to effectively address this issue. This study proposes an Attention-LSTM neural network model that performs deep learning on key input sequence information, so as to predict the distribution of emotional states in college students. By testing 60 consecutive days of emotional data, the model successfully predicts students' emotional distribution, triggers and resolution strategies, with an accuracy rate of no less than 99%. Compared with models such as ARIMA, SARIMA and VAR, this model shows significant advantages in accuracy, operational efficiency, and data collection requirements. The integration of deep learning technology with student management in this study offers a novel approach to address emotional issues among students under exceptional circumstances.

## Introduction

Since the COVID19 case was first reported in December 2019, China had quickly implemented regional lockdown measures, which effectively contained the spread of the virus within 3 months^1^. This fully demonstrates that "lockdown measures" can be regarded as a non-therapeutic measure that can effectively control the spread of the epidemic. Dr. Katherine. A of the United States once conducted a study that school closure contributed to the decrease in the incidence and mortality rate of COVID19 among students^2^. However, prolonged "lockdown measures" have concurrently triggered mental health issues among college students. The American Psychological Association has issued a psychological report on COVID19 during the pandemic, identifying young people aged 18–23 as a particularly vulnerable group. In this special period, about 25% of students feel severe anxiety^3^, 83% have experienced terrible situations, 26% have no access to mental health support, 44% have depressive thoughts, and 5% have suicidal thoughts^4^.

To address the physical and mental health issues faced by college students during the COVID19 pandemic, numerous scholars have conducted in-depth research on these matters. Erum Nadeem et al. used a community partner research framework to quickly evaluate the impact of COVID19 on teachers, students, and families^5^, However, the assessment model proposed in this research has a limited scope of applicability, and the model construction is highly complex. Natalie Förster et al. conducted a sample survey of German students to study the impact of the COVID19 pandemic on student performance^6^. The research results indicate that compared to localized data collection, the approach adopted is more representative and efficient. Tian Yan et al. applied the pre-trained Robustly Optimized BERT pre-training approach (RoBERTa) to learn text embedding from the Reddit messages, and leveraged the relational information among posted messages to train a graph attention network (GAT) for emotional prediction^7^. Fairoza Amira Binti Hamzah et al. visualized the official data and subsequently constructed a Susceptible-Exposed-Infectious-Recovered (SEIR) predictive model to forecast the public's health status under the COVID19 pandemic^8^. From the various studies mentioned by the scholars above, it is evident that in view of the uncertainty of the prevailing trend in the COVID19 pandemic, the ability to foresee the development trajectory of related issues in advance can proactively formulate the solutions and even take preventive measures to mitigate more severe consequences. Many researchers around the world incorporated various prediction techniques such as Susceptible–Infected–Recovered model, Susceptible–Exposed–Infected–Recovered model, and Auto Regressive Integrated Moving Average model (ARIMA) to forecast the spread of this pandemic^9^.

The predictive accuracy of the ARIMA model is relatively higher compared to the first two models, but it demands higher suitability of data. Therefore, Cia Vei Tan et al.^10^ developed the Seasonal AutoRegressive Integrated Moving Average (SARIMA) model to forecast the changing trends in the number of patients during the COVID19 pandemic. This model imposes lower requirements on the suitability of data, and the differences between predicted and observed values are mainly within a deviation range of 25%. Fabio Milani suggested the utilization of VAR (Vector AutoRegressive) models for forecasting the unemployment rates in specific regions during the COVID19 pandemic^10^. In the predictive model proposed by Farah Shahid and other scholars, it includes autoregressive integrated moving average (ARIMA)^12^, support vector regression (SVR)^13^and bidirectional LSTM (Bi-LSTM)^14^, which are used to predict the changes in confirmed cases and deaths in ten major countries affected by COVID19. This shows that it is feasible to use the appropriate model to predict and analyze student emotional states during the COVID19 epidemic.

This study primarily addresses the challenges faced by university students during the ongoing COVID19 pandemic, where prolonged confinement on campus can lead to emotional and psychological issues. To prevent such occurrences, early prediction of the emotional states of students^15^ and an investigation into the main causes of negative emotions within the student community are essential, alongside the identification of effective coping strategies. We propose employing Attention LSTM for in-depth learning on questionnaire survey data from students to predict future emotional states, primary triggers, and effective methods to alleviate negative emotions. Swift acquisition of relevant student data and the identification of effective coping strategies are crucial for universities and even governmental entities to promptly address psychological health issues during this unique period. The technical highlights of this study involve the integration of Attention LSTM with the LSTM model, suitable for handling sequential data and capturing temporal dependencies in survey data, thereby enhancing the understanding of emotional evolution. The Attention mechanism enables the model to focus on key information, improving sensitivity to important details. Additionally, it allows personalized adjustment of attention for different students, achieving more precise emotion prediction. In comparison to similar models, our approach exhibits significant advantages in terms of dataset collection, model complexity, and prediction accuracy. The broad significance of this research lies in its:

1. Introducing a novel approach that applies Attention LSTM to address collective mental health issues among students during the COVID19 pandemic, comparing its effectiveness against widely used predictive models to substantiate its superiority.
2. Enhancing the efficiency of addressing group-level psychological issues during exceptional circumstances by accurately forecasting the development of problems without systematically exploring the underlying causes. This is achieved by proposing a new method and predicting precise solutions based on existing data.
3. Against the backdrop of the normalized prevalence of the COVID19 pandemic, enriching student management data in higher education institutions to timely and effectively understand student needs. This approach provides valuable guidance for optimizing and enhancing student management practices.

## Results

### Prediction of mood change

During the peak period of the COVID19 epidemic, the proportion of college students with negative emotions showed a gradual increase. The emotional state of students was extremely unstable in the early stage of the school's "closed management" measures. In particular, the proportion of students with pleasant emotions and those with slightly negative emotions fluctuated significantly in the early stage. Overall, during the campus closure and epidemic prevention period, the emotional state of students gradually changed from its initial instability to stability. At the micro level, the number of students with pleasant emotions and those with slightly negative emotions decreases gradually, while the number of students with severely negative emotions continues to rise. Due to the large amount of samples and predictive information collected in the early stage, the initial data from September 15, 2022 to September 19, 2022 will be compared with the predicted data of the next five stable days. It is predicted that under the normalization of the epidemic, the proportion of negative emotions among college students will increase from approximately 30% to 70%. That is to say, COVID19 continues to affect the emotional state of college students, but there is a large difference in its influence before and after. The initial large fluctuations in students' emotional changes may be due to their unpreparedness for the sudden epidemic, so the related emotional data cannot be directly linked to students' mental health^16^. As time goes by, some of the reasons for negative emotions may change. Students may use their subjective initiative to resolve negative emotions, eventually making the emotions in a stable state.

**Figure 1:**
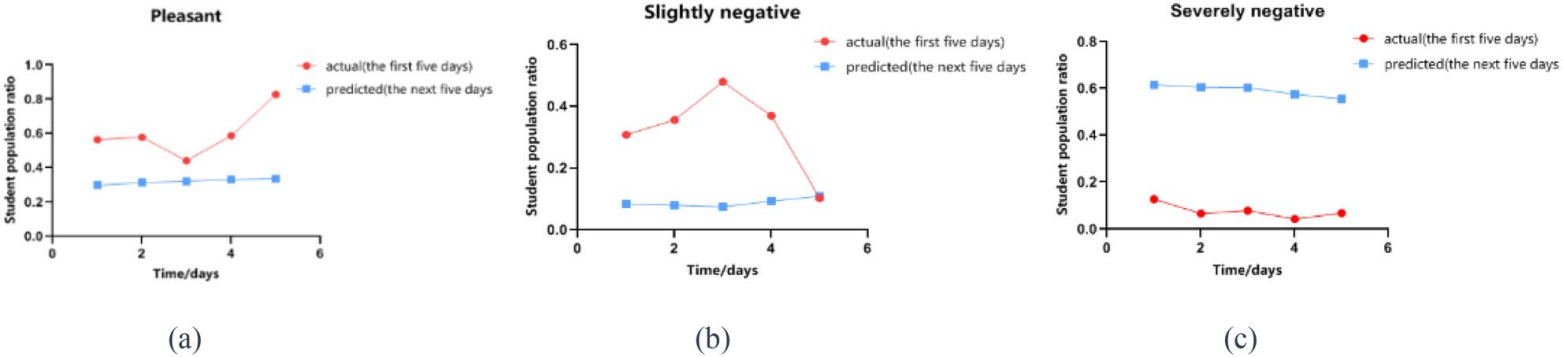
Comparison of the proportion of college students with pleasant, slightly negative and severely negative emotions. (Pleasant: Proportion of students with positive emotions; Slightly negative: Proportion of students with mildly negative emotions; Severely negative: Proportion of students with significantly negative emotions; actual: Actual collective emotional trends of students; predicted: Model-predicted collective emotional trends of students.).

Compared to the initial stage, the predicted proportion of students with pleasant emotions in the future will decrease by about 30% and then remain stable (a); the predicted proportion of students with slightly negative emotions will decrease by about 20% and remain stable (b); while the predicted proportion of students with severely negative emotions will increase sharply to 60% (c). From the data predicted by the Attention LSTM model in the next 5 days, it can be seen that the emotional state of college students is relatively stable. Approximately 30% of the students feel relaxed and happy, while about 60% of them have severely negative emotions. Only about 10% of students have slightly negative emotions in Fig. 1. This means that more than half of the college students are severely affected by COVID19, and this situation will not improve on its own unless the corresponding measures are taken.

### Emotional inducement prediction

**Figure 2:**
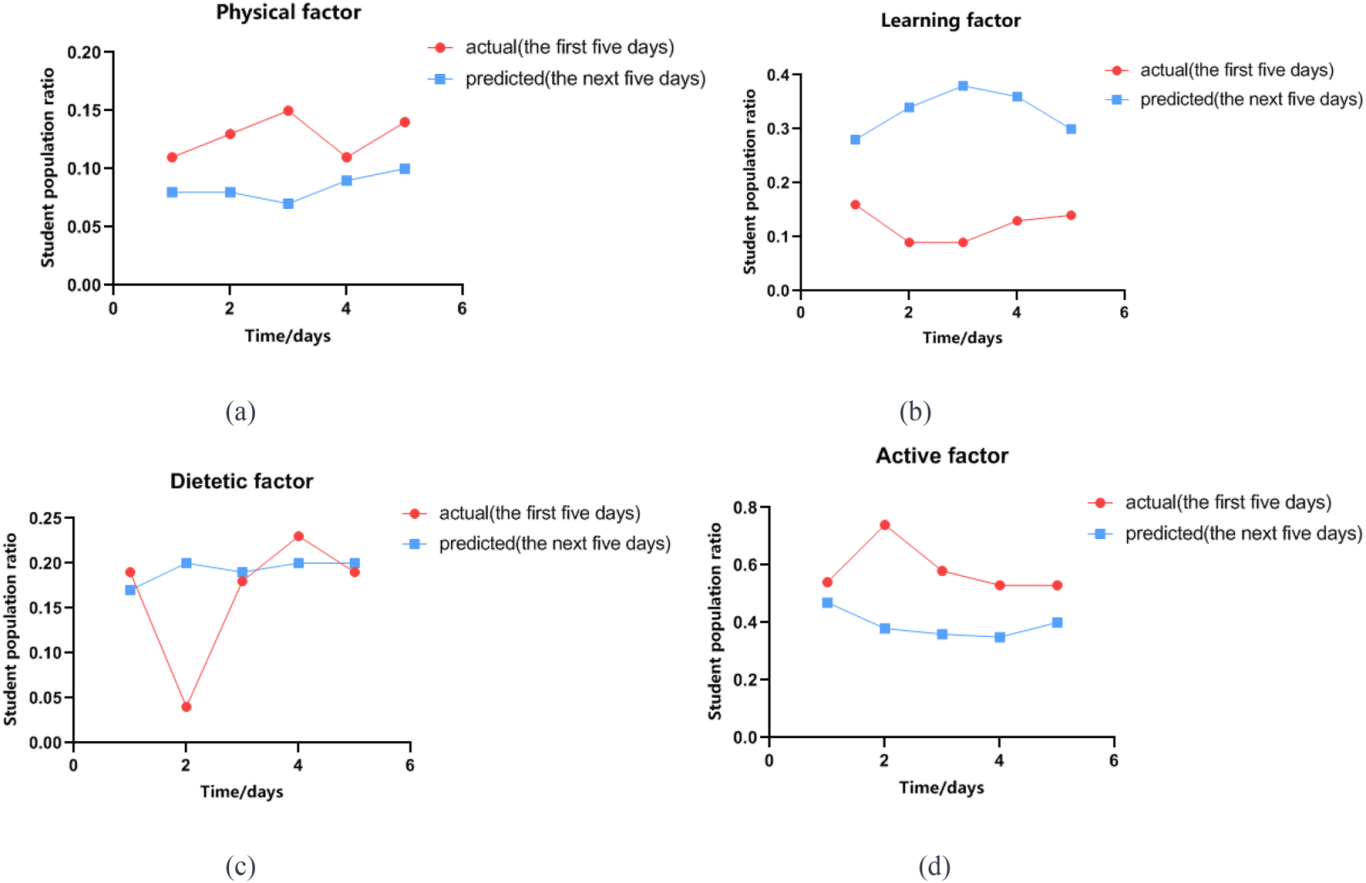
Changing trends of different emotional inducements. (Physical factor: The percentage of students who consider physical factors as the primary influencers of emotions; Learning factor: The percentage of students who consider learning factors as the primary influencers of emotions; Dietetic factor: The percentage of students who consider dietetic factors as the primary influencers of emotions; Active factor: The percentage of students who consider active factors as the primary influencers of emotions; actual: The actual trend of factors influencing the collective emotions of students; predicted: The trend of factors influencing the collective emotions of students predicted by the model.).

The conclusions of the investigation on emotional triggers indicate that the reasons for negative emotions among college students during the pandemic can be summarized into four aspects: physical factor, learning factor, dietetic factor, and active factor. The decrease in active factor before and after is the most obvious. In the initial stage of epidemic prevention and control, over 60% of students had negative emotions because they could not go out freely. However, the predicted data shows that the proportion of this trigger will continuously decrease over time, eventually stabilizing at around 40%. In contrast, the influence of learning factor on the emotions of college students is initially not obvious, accounting for only about 10% of the total. But predicted data shows that the proportion of students who believe that learning factor is the main cause of negative emotions will reach about 35%, ranking only second to active factor in Fig. 2.

Compared to the initial period, the predicted proportion of students regarding physical factor as the main cause of negative emotions is slightly lower, and then remains stable (a); the predicted proportion of students regarding learning factor as the main cause of negative emotions has significantly increased, even up to 40% (b); the predicted proportion of students regarding dietetic factor as the main cause of negative emotions has slightly increased and trends towards stability (c); and the predicted proportion of students regarding active factor as the main cause of negative emotions is slightly lower, but still accounts for a high proportion of 40% (d).

**Figure 3:**
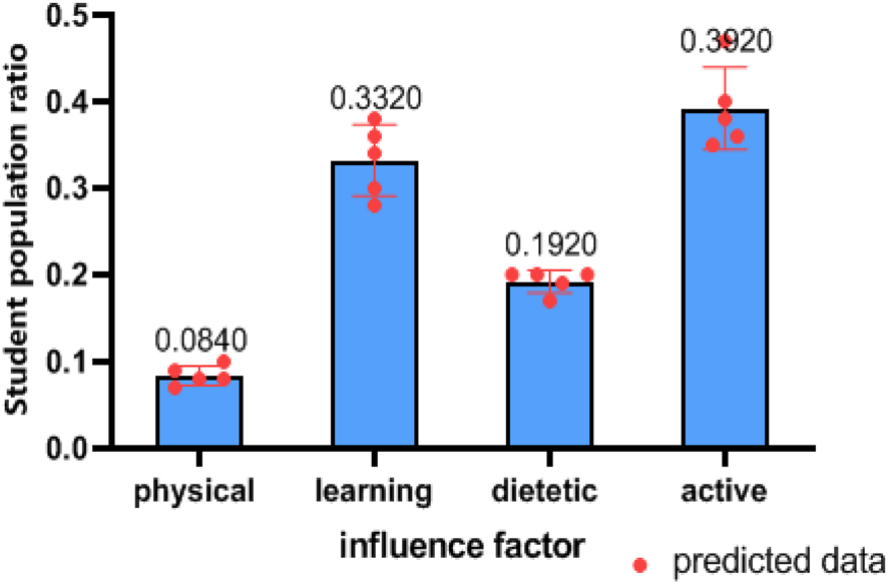
The proportion of predicted emotional inducements. (predicted data:the percentage distribution of students who consider physical factors, learning factors, dietetic factors, and active factors as the primary contributors influencing their emotional states.).

Predicting the main reasons affecting the emotional state of college students is helpful to provide a breakthrough for the school to take relevant measures. From the relatively stable predicted data, it can be seen that the physical factor has the smallest impact on students, which is only 8%. This indicates that the relatively closed campus management effectively blocked the spread of COVID19 on campus during this special period, and only some students have negative emotions because of their mental health. In contrast, the impact of active factor is the largest, reflecting the strong demand for outdoor activities. The long-term restriction of activities makes it difficult for college students to accept, but about 10% of students' demand for outdoor activities has decreased due to long-term space restrictions. They have adapted to living in a limited area. The learning factor is second only to the active factor, accounting for approximately 35%, and the predicted data is higher than that at the beginning. The main reason is that online learning is different from traditional face-to-face teaching. It not only requires higher autonomy and self-discipline of students, but also requires teachers to improve their teaching methods to adapt to online teaching. If students have difficulty adapting to online courses, the continuous accumulation of their psychological pressure is prone to induce negative emotions. The influence of dietetic factor is not significantly different before and after, and the final total proportion is stable at 20%. As Fig. 3 shows the catering supply of universities cannot meet the actual needs of college students without external supply.

### Response prediction

**Figure 4:**
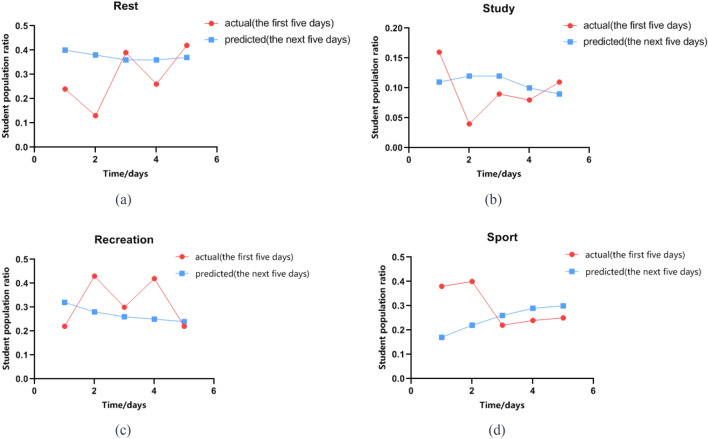
Changing trends of students' coping measures. (Rest: The percentage distribution of students who consider rest as the optimal means for emotional healing; Study: The percentage distribution of students who believe that studying is the best approach for emotional healing; Recreation: The percentage distribution of students who perceive engaging in recreational activities as the optimal method for emotional healing; Sport: The percentage distribution of students who consider participating in sports as the most effective way to alleviate emotions; Actual: The observed collective trends in students' strategies for emotional relief; Predicted: The anticipated trends in collective strategies for emotional relief forecasted by the model.).

Based on predictive analysis, under the normalization of the epidemic, the main ways for students to relieve stress in the future will involve rest, study, recreation and sport. Firstly, the proportion of students who use recreation as a way to relieve negative emotions is about 25%, with13% playing video games and 12% watching online videos. The number of people in each entertainment activity is relatively average. Secondly, there are also about 40% of students who use rest to relieve their emotions. However, as Fig. 4 shows the proportion of students who use self-study as an emotional coping measure is the lowest, only about 10%. This may be related to the closure of schools during the epidemic, unstable online learning environments, and the lack of offline learning. The unstable online learning environment has made it difficult for students to concentrate on learning, which has a negative impact on their learning outcomes. In addition, the panic and negative emotions brought about by the epidemic have also led to their lack of interest in learning, further affecting their learning status. Based on the comprehensive analysis of pre- and post-prediction data, we believe that engaging in restorative activities within the dormitory environment is an effective method to improve students’ negative emotional states. Nonetheless, it is crucial to note that an excessive prolongation of the rest period may not only exacerbate the negative emotional experiences encountered by students, but also lead to psychological disorders. A long-term mental illness trajectory analysis in six countries during the COVID19 pandemic showed that patients who stayed in bed for more than 7 days continued to have a higher risk of depression and anxiety symptoms^16^.

Predictions indicate that about 40% of students believe that rest is the most effective way to relieve negative emotions, and compared to the initial period, the proportion has increased significantly (a); about 10% of students believe that study is the most effective way to relieve negative emotions, and the number of students in this group remains relatively stable (b); about 25% of students believe that recreation is the most effective way to relieve negative emotions, but the number of students in this group is decreasing (c); about 25% of students believe that sport is the most effective way to relieve negative emotions, and the number of students in this group is constantly increasing (d).

### Data analysis

The proportion of negative emotions among college students during the COVID19 pandemic varies greatly, and it’s difficult to grasp the changing patterns. Introducing an Attention LSTM prediction model into the dynamic analysis of student emotions during the special period will help predict students' psychological state and enable schools to take preventive measures in advance^18^. We use the LSTM model to make the comparison between the predicted proportions of students with different emotions within the next 5 days and the actual ones, the results of which are shown in Tables 1, 2 and 3.

**Table 1:** Comparison analysis of the predicted and actual proportions of pleasant students.

Time	Student population ratio (actual)	Student population ratio (predicted)	R^2^
Day1	0.3020	0.3000	0.9998
Day2	0.3150	0.3140	
Day3	0.3220	0.3210	
Day4	0.3301	0.330	
Day5	0.3352	0.3350

**Table 2:** Comparison analysis of the predicted and actual proportions of slightly negative students.

Time	Student population ratio (actual)	Student population ratio (predicted)	R^2^
Day1	0.0839	0.0841	0.9983
Day2	0.0816	0.0803	
Day3	0.0757	0.0755	
Day4	0.0937	0.0936	
Day5	0.1098	0.1096

**Table 3:** Comparison analysis of predicted and actual proportions of severely negative students.

Time	Student population ratio (actual)	Student population ratio (predicted)	R^2^
Day1	0.6165	0.6159	0.9985
Day2	0.6060	0.6057	
Day3	0.6019	0.6035	
Day4	0.5761	0.5754	
Day5	0.5548	0.5554	

The calculation formula for correlation coefficient^18^ (Correl) is:

1 $$\text{Correl(X,Y) =}\frac{\sum (\text{x} - \overline{x})(\text{y} - \overline{y})}{\sqrt{{\sum (\text{x} - \overline{x})}^{2}\sum (y-\overline{y}{)}^{2}}}$$

$$ {\text{In}}\;{\text{this}}\;{\text{formula}},\;\overline{{\text{x}}} \;{\text{and}}\;\overline{{\text{y}}} \;{\text{are}}\;{\text{the}}\;{\text{sample}}\;{\text{means}}. $$

**Figure 5:**
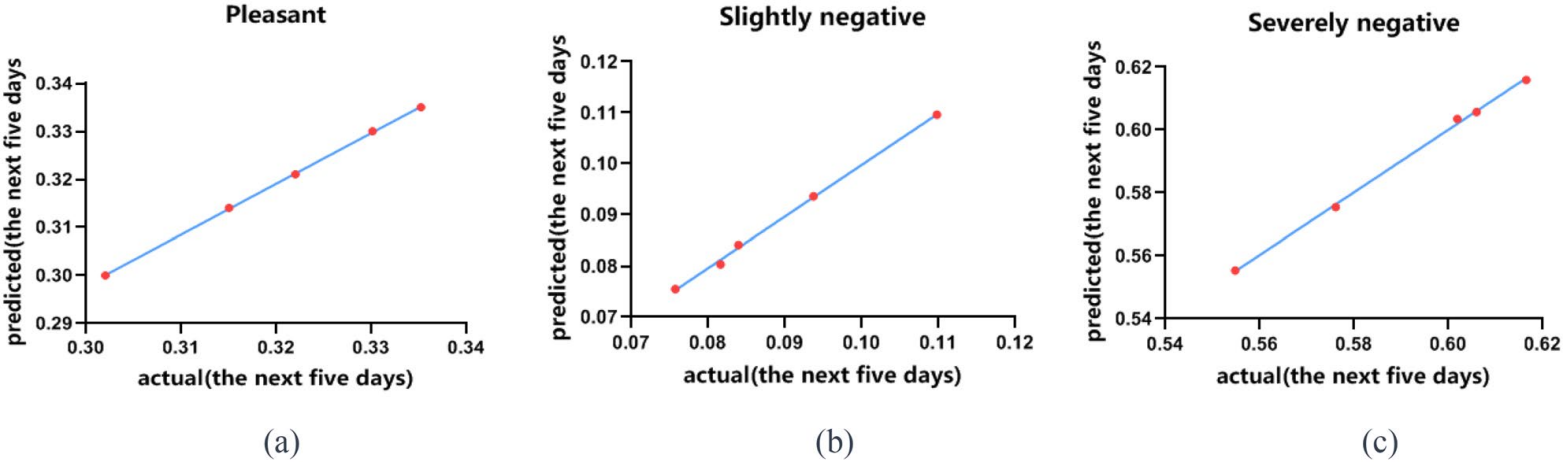
Comparison analysis of the predicted and actual proportion of pleasant students (**a**), slightly negative students (**b**) and severely negative students (**c**).

Using this formula, we found that the correlation coefficient between the predicted and actual proportions of students with a positive mood was 0.9998, the coefficient for students with slightly negative emotions was 0.9983, and the coefficient for students with severely negative emotions was 0.9985. As the correlation between the predicted and actual results of three emotions is extremely high, it can be concluded that the Attention LSTM prediction model is highly compatible with the emotional changes of university students during the COVID19 pandemic. The model can accurately predict future changes of various emotional indicators through limited data, as shown in Fig. 5.

In order to analyze whether Attention LSTM exhibits a notable advantage in predictive accuracy compared to Traditional Psychological Theoretical Model, ARIMA, SARIMA, and VAR, as mentioned by other scholars, the "Students with Severe Negative Emotions" dataset is applied to all models. The predictive accuracy is evaluated using the Pearson correlation coefficient analysis method, as illustrated in Table 4.

**Table 4:** Comparison of correlation coefficients under different predictive models.

Model	Traditional psychological theoretical model	ARIMA	SARIMA	VAR	Attention LSTM
R2	0.6975	0.8325	0.8563	0.8753	0.9985

Through horizontal data analysis, it is evident that utilizing different types of prediction models for forecasting datasets related to "students with severe negative emotions" yields prediction data. The closer the correlation coefficient between the obtained prediction data and the actual data is to 1, the higher the corresponding model's applicability accuracy. Table 4 lists the correlation coefficient values corresponding to different models. The correlation coefficient for the Traditional Psychological Theoretical Model is only 0.6975, indicating the lowest accuracy. In contrast, the Attention LSTM model employed in this study exhibits a correlation coefficient of 0.9985, signifying the highest accuracy.

## Discussion

The most commonly used model for predicting changes in human emotional state in early stages was the traditional psychological theoretical theory model, which is mainly based on the self-determination theory. This theory only focuses on the internal motivation of the predicted object, attempting to analyze the difference between an individual's internal and external motivations and focusing on the dominance of internal motivation in an individual's behavior and choice. This model can only perform single-line prediction, that is, if the emotional changes of university students are all triggered by internal factors, the psychological theory model is reliable and can predict the future direction of students' emotions by summarizing a large amount of psychological data with time as an ordered variable. However, the factors that affect student emotions are diverse, so the accuracy of using this model for prediction is not high. In comparison, models based on statistical methods are more reasonable. Mainstream models that can be used for predicting student emotions include the ARIMA model, SARIMA model, VAR model, etc.

The ARIMA model is a stationary time series prediction model based on linear regression, which includes three parts: auto-regression (AR): a model that uses the dependency relationship between observed values and their lagged values; integration (I): a method of differencing the observed values from the previous time lagged observations to make the time series stationary; and moving average (MA): a model that uses the dependency relationship between the error terms and the previous ones. The model requires the original data to have a stable time series and to be differenced to remove trends and seasonality^20^. The average relative error in the prediction results can generally be controlled at around 10%. The SARIMA model is a seasonal extension of the ARIMA model, which transforms non-smooth time series into smooth cyclical sequences. As this model can not only analyze the irregular non-smooth time series data, but also consider the seasonal characteristics in time series data, it can be applied to the models with medium or even longer time series. This model has higher accuracy in predicting the data that takes seasonality and periodicity as influencing factors. The average relative error in the prediction results is slightly lower than that of the ARIMA model. The VAR model adopts the form of multi-equation, eliminates many assumptions in traditional models, includes lagged terms, and estimates the influence between variables in the model. Since it can consider the mutual influence between multiple time series, the VAR model can usually make the predictions more accurately when multiple variables being considered simultaneously. If the relationship between different variables (such as learning status, social activities, diet and health) is considered, the VAR model may be more suitable for predicting students’ emotions. Although the models based on psychological theory require less data, they require strong professional theoretical support. The models based on statistical methods have higher prediction accuracy, but they require manual selection and tuning, which is relatively cumbersome.

Traditional psychological theoretical Model: Explains human behavior based on psychological principles; ARIMA: Time series model for forecasting; SARIMA: Extends ARIMA for seasonal patterns in data; VAR: Multivariate time series forecasting model.

This study employed various forecasting models for experimental analysis of student emotional data, as illustrated in Table 5. The ARIMA model, a relatively straightforward model, exhibits high operational efficiency, particularly suited for modeling and predicting short time series. However, ARIMA may perform inadequately when dealing with complex nonlinear and seasonal time series. Secondly, the SARIMA model, an extension of ARIMA, incorporates seasonal components. Despite its higher model complexity and the need to select appropriate seasonal orders, SARIMA demonstrates improved prediction performance, particularly when handling data with seasonal considerations, surpassing ARIMA. The VAR model, a multi-equation model, is applicable when there are relationships among multiple variables. While offering comprehensive modeling capabilities, VAR exhibits higher model complexity and relatively lower operational efficiency when dealing with extensive multivariate time series data. Compared to other models, Attention LSTM has the following advantages^21^: (1) Attention LSTM can better capture sequential information, as it has memory units and forget gates that can effectively handle long-term dependencies in the sequence. (2)The Attention mechanism can help the model automatically learn emotional features, which has a good effect on emotion recognition and prediction tasks. Compared to other models, Attention LSTM can more accurately identify changes and trends in student emotions. (3) Thanks to the Attention mechanism, Attention LSTM can generate an attention distribution for each time step, helping us understand how the model predicts the emotional state at each time step. As shown in Table 4, Attention LSTM can also generate visual results, which can better help schools understand the emotional state of students.

**Table 5:** Comparison of different models in student emotion prediction.

Model	Traditional psychological theoretical model	ARIMA	SARIMA	VAR	Attention LSTM
Data	Non-linear	Linear	Linear	Linear	Non-linear
	Stationary	Stationary	Stationary	Stationary	Non-stationary
	Non-periodic	Non-periodic	Periodic	Non-periodic	Non-periodic
Computation	Very complex	General complex	General complex	Relatively complex	Very complex
Operational efficiency	Lower	Relatively efficient	Relatively low	Relatively low	Relatively low
Prediction	Inaccuracy	Low accuracy	Low accuracy	High accuracy	High accuracy

## Methods and materials

### Methods

**Figure 6:**
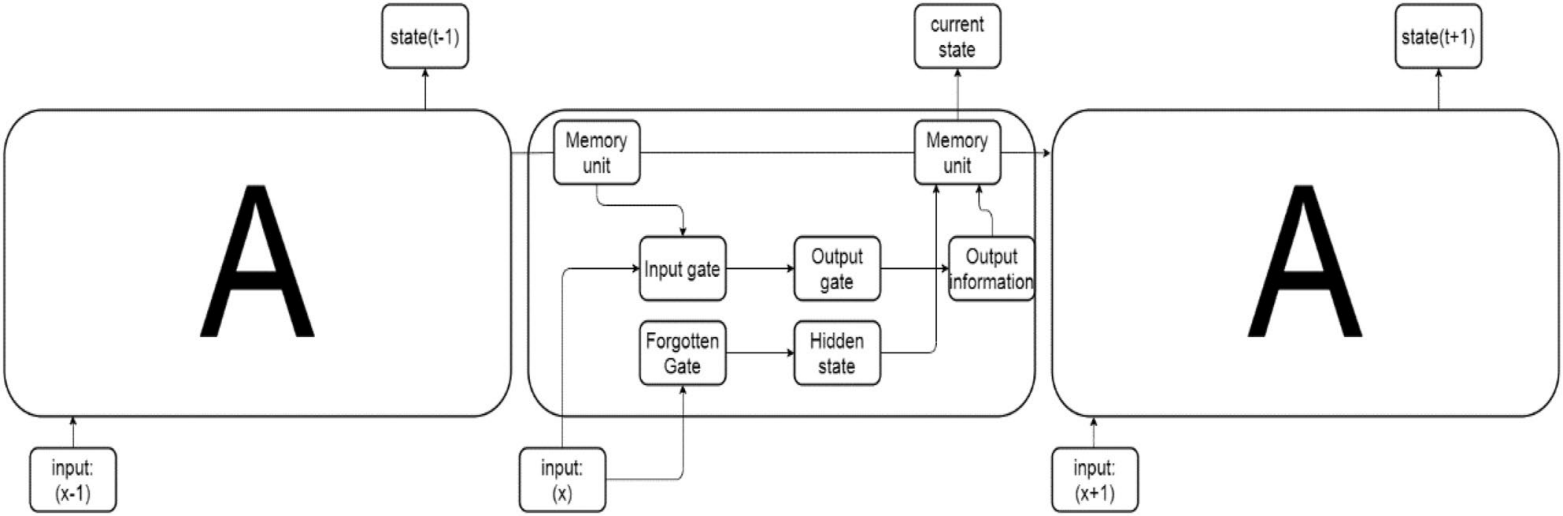
Schematic Diagram of LSTM Model. (Input: Represents input sequences; Memory Unit: Internal memory handling information transfer; Input Gate: Controls input information flow; Forget Gate: Determines forgotten information; Output Gate: Decides output information; Hidden State: Output capturing current understanding; Output Information: Model's final output.).

This study employs an empirical analysis approach, utilizing survey questionnaires for the collection of emotional data from university students during the COVID19 pandemic. One-Class SVM^21^ and K-Nearest Neighbors^22^ are employed for data cleaning to reduce dataset uncertainty, transforming it into analytically viable data. As Claude Shannon, the founder of information theory, stated, "When information is viewed as a measure of order and negentropy, it is necessary to reduce uncertainty." As illustrated in Fig. 6, LSTM is a recurrent neural network structure with notable advantages in processing and predicting time-series data^23^. Given that the collected questionnaire data in this study are time-series with long-term dependencies, the LSTM (Long Short-Term Memory) model is deemed suitable for analysis^24^.

This study employs deep learning through LSTM model training with an effective dataset to predict long-term emotional trends and effective coping strategies among university students^25^. The rationale lies in elucidating future psychological state changes based on phenomenological conclusions regarding emotional states. Causal and strategic predictions are utilized to guide issue resolution. Furthermore, strategic predictions help identify current inadequacies in addressing collective emotional challenges among university students.The experimental protocols are approved by the Ethics Committee of Fujian Chuanzheng Communications College. All methods are performed in accordance with the relevant guidelines and regulations.

### Data collection

The target population of this study comprises all current university students in Fujian Province. With a total student population exceeding 900,000, a comprehensive survey is impractical. The research selects a few representative colleges and universities as samples, striving to include a diverse range of associate, undergraduate, graduate, and doctoral students. To ensure representative participation, a stratified sampling approach^23^ is employed based on academic levels within each chosen institution. The candidate sample schools, including Fuzhou University and Fujian Merchant Marine Academy, allocate 1000 survey slots each, with proportional reallocation for undergraduate, master's, and doctoral students. Data collection primarily relies on the student management systems of each institution to distribute survey questionnaires, urging participating students to truthfully respond. The study's target group positioning and data collection approach are scientifically designed. By considering the diversity of universities in Fujian Province and selecting four distinct types of institutions, encompassing comprehensive universities, specialized colleges, and vocational technical colleges, the study ensures operational feasibility, sample diversity, and relative balance in weighting during data collection. All data samples used in this study have obtained the informed consent of the participants and the relevant legal guardians.

### Questionnaire design

This study is specifically designed to survey the emotional states of on-campus students during the unique period of the COVID19 pandemic. The questionnaire distribution period spans from August 26, 2022, to November 14, 2022.the peak period of the COVID19 outbreak in Fujian Province. The faster the spread of COVID19 in society, the greater the impact it has on college students, and the more obvious the emotional fluctuations it causes. The design of the questionnaire relies on the most commonly online survey tool in China–sojump^11^. The survey questions mainly focus on three aspects: the current emotional state of college students, direct or indirect negative emotional triggers, and effective coping strategies adopted by students. The total amount of questions involved is 22. In order to intuitively reflect the changes in students' emotional indicators during the survey period, a new survey needs to be generated every day and completed on the same day. As of November 14th, 2022, we had collected a total of 60 valid survey questionnaires. These questionnaires are audited and proofread to ensure that the data of each questionnaire covers four representative universities, with a capacity of no less than 4000 students. The survey questionnaire was verified through the Split-Half Method. After applying the Spearman–Brown correction formula, the reliability coefficient was 0.91, indicating high reliability and effectiveness. To ensure that the dataset collected by the questionnaire is consistent with the LSTM model for accurate predictions, an initial test is conducted using a subset of psychology class A students as a validation sample. The predicted emotional state data for the sample students is closely consistent with the actual situations, confirming that the questionnaire is suitable for LSTM model.

### Model design

**Figure 7:**
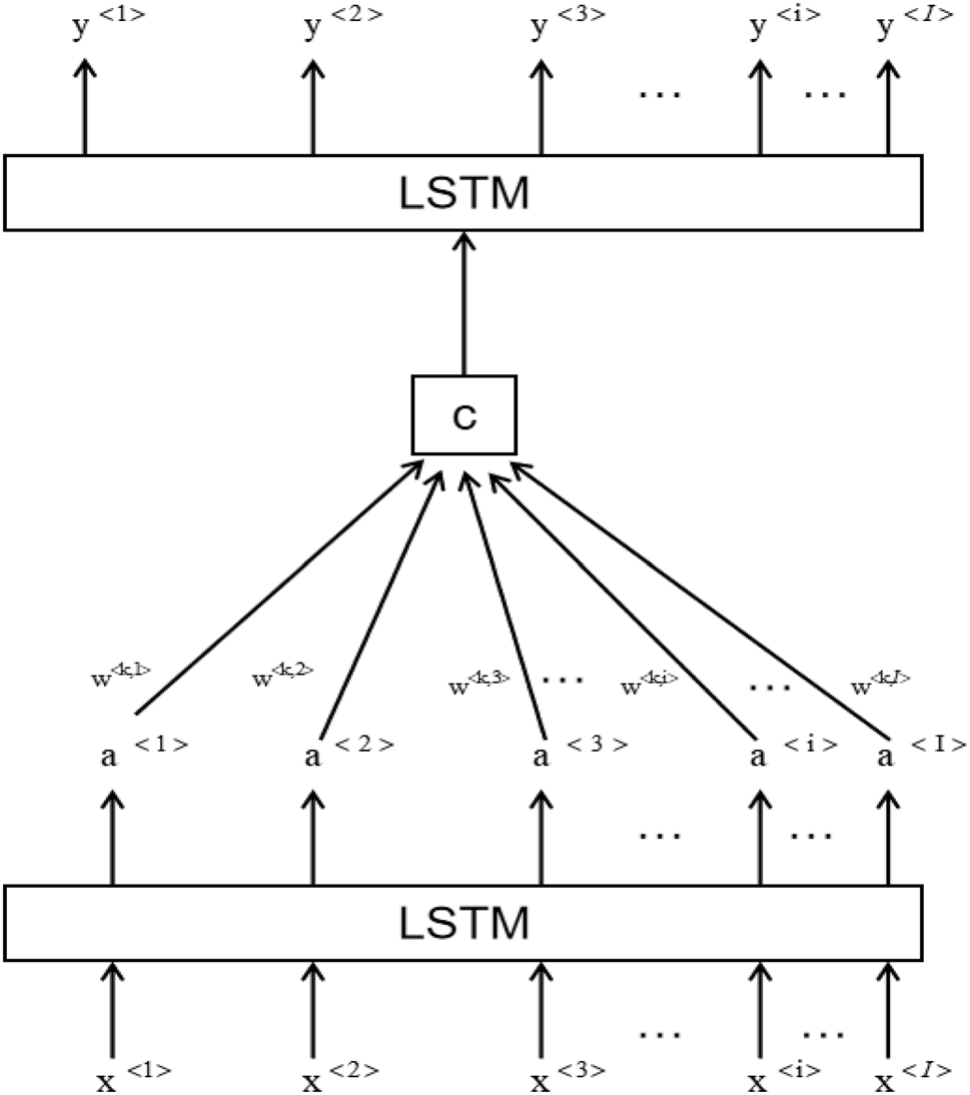
LSTM network structure based on the attention mechanism.

This study adopts a LSTM network structure based on the attention mechanism^12^. In order to better learn the information in the input sequence, the attention mechanism simulates the human attention model, and allocates more attention to the key parts in the input sequence that influence the output results. We use the attention mechanism as the interface between two LSTM networks. Firstly, an LSTM network is used to process the input sequence to achieve high-level feature learning. Secondly, by allocating attention weights reasonably, we achieve the memory unit solving. Finally, another LSTM network in Fig. 7 is run to achieve ultra-short-term load forecasting.

As shown in Fig. 7, this is an encoder-decoder model, Seq2Seq model. X^<i>^ represents the input sequence; y^<i>^ represents the output result; a^<i>^ represents the learned features of the input sequence x; w^<k,i>^represents the attention weight of each feature, indicating the influence of the t-th feature on the result y^<k>^, that is, the attention on a^<i>^ at time y; and c is the memory unit.

The formula for calculating the attention weight w^<k,i>^ is:

2 $$ w^{{\left\langle {k,i} \right\rangle }} = \frac{{\exp \left( {e^{{\left\langle {k,i} \right\rangle }} } \right)}}{{\sum\nolimits_{i = 1}^{I} {\exp \left( {e^{{\left\langle {k,i} \right\rangle }} } \right)} }} $$

The mean squared error (MSE) of loss function is used to reflect the degree of difference between the estimated value and the estimated value, and is used as the target loss function of this network. Its calculation formula is:

3 $$ MSE(\mathop \theta \limits^{ \wedge } ) = E(\mathop \theta \limits^{ \wedge } - \theta )^{2} $$

In this formula: *θ* is the estimated value of the parameter; and *θ* is the actual value of the parameter.

**Figure 8:**
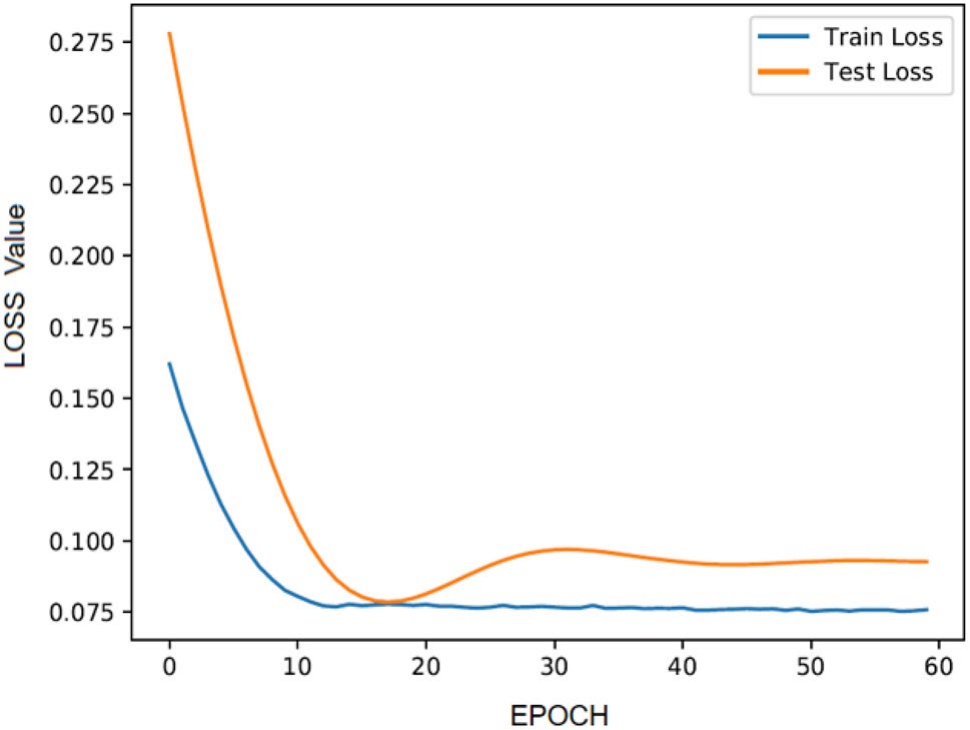
Training and validation loss gradient diagram. (Train Loss: A scalar value calculated through a loss function during model training is used to measure the model's performance on the training data; Test Loss: The loss value computed by a test dataset after the model has been trained.).

Adam algorithm is used to replace the traditional stochastic gradient descent process. Based on training data and loss function, the first and second moment estimates of each parameter are calculated. In accordance with the calculated results, the learning rate of each parameter is dynamically adjusted to achieve iterative updates of network weights. In the training process, we use a learning rate (Ir = 0.01) and train the parameters for 5 M iterations. The training gradually decreases in an exponential manner and determines the optimal solution in the later stage. After 60 rounds of training, we select the best optimization result. The loss gradient obtained from the training in this study is shown in Fig. 8.

## Conclusion

In this study, Attention LSTM was used to predict the emotional change trend of college students during the epidemic of COVID19, and accurate data on the distribution of students 'emotional types were obtained. The results show that students' emotional state gradually converges from high discrete fluctuations to relatively stable negative states. Afterwards, the students who are more susceptible to the prolonged impact of COVID19 begin to differentiate gradually, with nearly 70% of them staying in negative emotions for a long time. By comparing the Attention LSTM model proposed in this study with other models, it can be concluded that when solving the collective mental health problems of college students in special periods, using Attention LSTM for predictive analysis after data collection through questionnaires is efficient and accurate, with an accuracy of no less than 99%.Therefore, the model can provide valuable reference ideas for effectively solving students' emotional problems and optimizing the management work. In the practical application of student management, on the one hand, we can directly solve the future problems according to the proportion of predicted influencing factors. That is to say, the future development trend, relevant causes and effective solutions can be directly inferred from the occurrence of student problems, although the causal relationship between the three is not yet clear. On the other hand, through the analysis of the predicted data, we can dig out the real psychological needs of students, and reversely deduce the logical relationship between various links, so as to improve the quality and efficiency of student management.

However, the method proposed in this study still has certain limitations, especially in sample data collection. For instance, the incompleteness of the collected data may lead to a decrease in the model's generalization ability, and sample selection bias might result in insufficient representation of the entire college students. To address the limitations of this research, several future research directions are suggested: (1) Improvement in Data Collection Methods, as well as the Sample Size and Representativeness: Enhance data collection methods to mitigate issues related to data incompleteness and expand both the size and representativeness of the sample. (2) Further Exploration of Deep Learning Technologies: Investigate advancements in deep learning technologies to enhance the model's performance and robustness. This may involve optimizing model architecture, incorporating more sophisticated attention mechanisms, and employing improved model tuning methods. (3) Thoughts on Model Fusion: Explore the integration of the Attention-LSTM model with other types of models to leverage their respective strengths and enhance the overall predictive performance.

### Supplementary Information

Supplementary Information.

## Data Availability

The datasets generated and analyzed for the current study are not publicly available due to the confidentiality policies and laws of the country. But they are available from the corresponding author upon reasonable request.

## Code availability

For reasons of confidentiality and compliance with relevant legal, the code used in the current study is not publicly available. However, interested parties may request access to the code from the corresponding author, and reasonable requests will be considered.
