# Supplementary material for: The emotion prediction of college students with attention LSTM during the COVID19 epidemic
Source: Sci Rep. 2023 Dec 20;13:22825. doi: 10.1038/s41598-023-50322-x (PMC10739690; doi:10.1038/s41598-023-50322-x)
Supplement: Supplementary file 1 — Supplementary Information. [file 41598_2023_50322_MOESM1_ESM.docx]

The Emotion Prediction of College Students with Attention LSTM during the COVID19 Epidemic

**Mengwei Wu^1^,Shaodan Lin ^1*^,Chenhan Xiao ^1^,Xiulin Xiao ^1^,Siwei Xu^1^,Shuhan Yu ^1^**

(^1^*College of Mechanical and Intelligent Manufacturing, Fujian Chuanzheng Communications College, Fuzhou 350007, China.*email:linshaodan66@qq.com.*)

**Survey on the Living Conditions of College Students During the Pandemic Campus Closure**

1.Your gender: Male/Female

2.Your current academic year: 2022/2021

3.After a period of pandemic-related campus closure, how would you describe your current emotional state? Very happy and relaxed/Accustomed with no specific feelings/Slightly negative emotions/Noticeably negative emotions

4.What do you think is the main cause of your negative emotions? Poor physical condition/Inability to adapt to the pace of online learning/ Dissatisfaction with current accommodation and dining conditions/Inability to go outdoors due to long-term campus confinement

5.What methods will you primarily use to alleviate negative emotions? Sleeping and resting/Reading and studying/Using electronic devices for entertainment/Engaging in outdoor sports and exercise

6.How much time do you spend on electronic devices for entertainment today? Less than 3 hours/3-6 hours/6 hours or more

7.What entertainment activities do you engage in using electronic devices? Playing games/Watching videos/Engaging in online social activities/Other (Specify)

8.How would you describe the atmosphere in your current dormitory? Harmonious atmosphere, frequently participating in collective dormitory activities/Normal atmosphere, generally busy with individual tasks/A tense atmosphere due to conflicts and friction among roommates from prolonged cohabitation

9.How is your current physical condition? Very healthy/Experiencing symptoms of fever/Experiencing symptoms of a cold/Experiencing symptoms of diarrhea/Experiencing symptoms of skin infection/Slightly injured

10.Is your current daily routine regular? Very regular/Somewhat irregular/Very irregular

11.How long do you sleep per day? Less than 6 hours/6-8 hours/8 hours or more

12.Did you engage in physical exercise today? Yes/No

13.How long did your physical exercise last today? 2 hours or more/About 1 hour/Less than 0.5 hours/No exercise

14.What physical exercise activities did you engage in today? Running/Playing sports/Indoor fitness in the dormitory/None

15.Are you adapting to current online course teaching? Yes/No

16.How long was your online class today? No class or 1-2 classes/3-4 classes/4 classes or more

17.How disciplined were you in today's online class attendance? Very disciplined/Moderately disciplined/Not disciplined at all

18.How effective was your classroom learning today? Completely grasped the knowledge taught by the teacher/Partially grasped the knowledge taught by the teacher/Did not grasp the knowledge taught by the teacher

19.Did you complete the assignments assigned by the teacher on time and in the required amount today? Yes/No

20.How would you evaluate the quality of today's online classes? Very satisfied/Fairly satisfied/Not satisfied

21.How did you overcome difficulties encountered during online learning today? Asking questions online/Browsing the internet for information/Avoiding the problem

22.Do you currently have any urgent issues that need to be addressed?
